# Rcf1 Modulates Cytochrome *c* Oxidase Activity Especially Under Energy-Demanding Conditions

**DOI:** 10.3389/fphys.2019.01555

**Published:** 2020-01-14

**Authors:** Hannah Dawitz, Jacob Schäfer, Judith M. Schaart, Wout Magits, Peter Brzezinski, Martin Ott

**Affiliations:** Department of Biochemistry and Biophysics, Stockholm University, Stockholm, Sweden

**Keywords:** Rcf1, Rcf2, respiratory supercomplex, cytochrome *c* oxidase, *bc*_1_ complex, interaction partners, *Saccharomyces cerevisiae*

## Abstract

The mitochondrial respiratory chain is assembled into supercomplexes. Previously, two respiratory supercomplex-associated proteins, Rcf1 and Rcf2, were identified in *Saccharomyces cerevisiae*, which were initially suggested to mediate supercomplex formation. Recent evidence suggests that these factors instead are involved in cytochrome *c* oxidase biogenesis. We demonstrate here that Rcf1 mediates proper function of cytochrome *c* oxidase, while binding of Rcf2 results in a decrease of cytochrome *c* oxidase activity. Chemical crosslink experiments demonstrate that the conserved Hig-domain as well as the fungi specific C-terminus of Rcf1 are involved in molecular interactions with the cytochrome *c* oxidase subunit Cox3. We propose that Rcf1 modulates cytochrome *c* oxidase activity by direct binding to the oxidase to trigger changes in subunit Cox1, which harbors the catalytic site. Additionally, Rcf1 interaction with cytochrome *c* oxidase in the supercomplexes increases under respiratory conditions. These observations indicate that Rcf1 could enable the tuning of the respiratory chain depending on metabolic needs or repair damages at the catalytic site.

## Introduction

Mitochondria exert a plethora of metabolic functions including the TCA cycle, β-oxidation of fatty acids, heme synthesis, calcium storage and oxidative phosphorylation (OXPHOS) (Spinelli and Haigis, 2018). ATP is the product of the OXPHOS system, a system of complexes residing in the inner mitochondrial membrane consisting of the respiratory chain complexes and the ATP synthase. Electrons are transferred from electron donors like NADH and FADH_2_ through the different complexes of the respiratory chain, which maintain a proton gradient by translocating protons across the inner mitochondrial membrane. The ATP synthase utilizes this proton gradient to generate ATP from ADP and P_*i*_. Understanding of the working mechanisms of the respiratory chain is especially important considering the diseases associated with a dysfunctional respiratory chain (Barrientos et al., 2002; McKenzie et al., 2006; Picard et al., 2016).

In the yeast *Saccharomyces cerevisiae* the respiratory chain consists of complexes II, III, and IV. Complex III (*bc*_1_ complex) and Complex IV (cytochrome *c* oxidase) assemble to respiratory supercomplexes (III_2_IV and III_2_IV_2_), structures which persist in mild detergent conditions (Cruciat et al., 2000; Schägger, 2000). The function of respiratory supercomplexes is debated with proposals ranging from substrate channeling over a decrease in ROS to avoiding aggregation of proteins in the crowded inner mitochondrial membrane (IMM) (Genova and Lenaz, 2014; Milenkovic et al., 2017; Fedor et al., 2018; Lobo-Jarne and Ugalde, 2018). Several proteins were found to associate with the respiratory supercomplexes without being a subunit of the individual complexes, namely Coi1, Aac2, Rcf1, and Rcf2 (Claypool et al., 2008; Dienhart and Stuart, 2008; Chen et al., 2012; Strogolova et al., 2012; Vukotic et al., 2012; Singhal et al., 2017). Recent cryo-EM structures of the yeast supercomplexes could not resolve these proteins interacting with the complexes (Letts et al., 2016; Hartley et al., 2019; Rathore et al., 2019). Two of these proteins, respiratory supercomplex factors 1 and 2 (Rcf1 and Rcf2), are part of the Hig-domain protein family. They share a high sequence similarity in this domain and are homologs to mammalian HIGD1A and HIGD2A. While HIGD1A and HIGD2A are expressed under stress conditions like hypoxia or low glucose levels (Wang et al., 2006; Ameri et al., 2013; Hayashi et al., 2015; Salazar et al., 2019), Rcf1 and Rcf2 are constitutively expressed (Garlich et al., 2017). The association of Rcf1 and Rcf2 as well as HIGD1A and HIGD2A with the respiratory supercomplexes has been described previously (Chen et al., 2012; Strogolova et al., 2012; Vukotic et al., 2012; Hayashi et al., 2015). Two studies independently showed that Rcf1 interacts with the cytochrome *c* oxidase subunit Cox3 during assembly (Su et al., 2014; Garlich et al., 2017). Nevertheless, the exact function of the proteins is still under debate.

Here, we show that Rcf1 and Rcf2 regulate the respiration of *S. cerevisiae* by modulating the activity of cytochrome *c* oxidase without strongly affecting supercomplex assembly and activity. Furthermore, we show that Rcf1 and Rcf2 are not stoichiometric subunits of cytochrome *c* oxidase. Our data suggest that they rather act as modulators of cytochrome *c* oxidase activity especially under energy demanding conditions. We confirmed the interaction partner of Rcf1 to be Cox3 and established that not only the conserved Hig-domain of Rcf1 is part of the interaction but also the fungi-specific C-terminus. Furthermore, these interactions are established during cytochrome *c* oxidase assembly, as published previously (Garlich et al., 2017), but are maintained in monomeric cytochrome *c* oxidase and supercomplexes. We propose that Rcf1 modulates cytochrome *c* oxidase activity by changing the environment of the active site or by repairing damages occurring during the catalytic cycle.

## Results

### Rcf1 and Rcf2 Affect Respiratory Ability and Supercomplex Assembly

**FIGURE 1:**
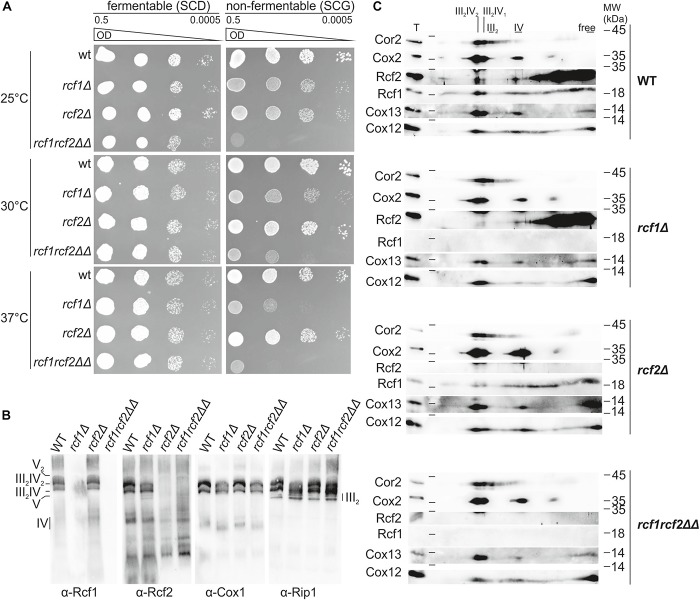
Rcf1 and Rcf2 affect respiratory growth but influence supercomplex assembly only slightly. **(A)** A 10-fold serial dilution growth assay on fermentable and non-fermentable medium at different temperatures showed that absence of Rcf1 decreases respiratory ability of the strains. The absence of Rcf2 showed only in combination with loss of Rcf1 a strong decrease in growth. **(B)** Mitochondria were purified from cells grown to logarithmic phase in YPGal (30°C). Supercomplex assembly of mitochondria solubilized in 2% digitonin was analyzed by BN-PAGE followed by subsequent Western blot and immuno decoration showing supercomplex assembly in the absence of Rcf1 and Rcf2. The antibody against Rcf2 shows unspecific binding in the lower molecular weight range. **(C)** Respiratory chain complex assembly of mitochondria purified from cells grown in YPGal (logarithmic phase, 30°C) was analyzed. Mitochondria were solubilized in 2% digitonin, separated by BN-PAGE followed by separation on 2D polyacrylamide gels and Western blot analysis. Rcf1 and Rcf2 comigrate with supercomplexes. Upon loss of Rcf1, Rcf2 shifts mostly out of the supercomplexes. Still Rcf1 and Rcf2 can associate with supercomplexes in the absence of the respective other protein. Supercomplexes can still assemble in the absence of Rcf1, Rcf2 or both proteins combined although in a lower amount. T, total; III_2_IV_2_, III_2_IV, forms of supercomplexes; III_2_, dimer of *bc*_1_ complex; IV, cytochrome *c* oxidase.

Rcf1 and Rcf2 belong to the conserved Hig-domain protein family. We investigated the effect of the loss of Rcf1 and Rcf2 on respiratory ability under normal and under stress conditions using growth assays (Figure 1A). Under normal conditions (30°C), lack of Rcf1 led to a mild growth defect on the non-fermentable carbon source glycerol. The same phenotype was observed at mild cold stress (25°C), while a stronger growth defect was observed under mild heat stress (37°C). In contrast, loss of Rcf2 did not impact growth at normal and heat shock conditions. Only at 25°C a slight decrease in growth could be observed. Surprisingly, loss of both Rcf1 and Rcf2 induced a strong growth defect already under normal conditions and a more severe growth defect under stress conditions (25 and 37°C, respectively). Thus, Rcf1 is important for the respiratory ability of *S. cerevisiae*, while Rcf2 affects respiration only in absence of Rcf1.

To investigate how Rcf1 and Rcf2 affect respiration, we analyzed the assembly of the respiratory chain. In yeast, the *bc*_1_ complex (III) and the cytochrome *c* oxidase (IV) form respiratory supercomplexes, consisting of an obligate *bc*_1_ complex dimer and one or two copies of cytochrome *c* oxidase (III_2_IV and III_2_IV_2_). Analysis of supercomplex formation by BlueNative-PAGE (BN-PAGE) with subsequent Western blot and immuno decoration showed that Rcf1 and Rcf2 are part of the supercomplexes (Figure 1B). However, respiratory supercomplexes formed in the absence of either Rcf1 or Rcf2 as well as in the absence of both proteins. Furthermore, Rcf1 and Rcf2 remained in the supercomplexes in the absence of the respective other protein. To investigate the supercomplex formation in more detail we resolved complexes on a 2D polyacrylamide gel followed by Western blotting and immuno decoration (Figure 1C). Decoration against Cor2, as a representative for the *bc*_1_ complex, showed free *bc*_1_ complex dimer (III_2_) as well as roughly equal amounts of both forms of supercomplexes. Cox2, a core subunit of cytochrome *c* oxidase, as well as the accessory subunits Cox12 and Cox13 were comigrating with the supercomplexes and monomeric cytochrome *c* oxidase. Since the supercomplex III_2_IV_2_ contains two copies of cytochrome *c* oxidase, the signal for all three cytochrome *c* oxidase subunits was more intense in this supercomplex form. Rcf1 and Rcf2 comigrated with the supercomplexes showing more intense signal in the higher supercomplex compared to the lower, suggesting that they primarily interact with cytochrome *c* oxidase. Likewise, both proteins comigrated also with monomeric cytochrome *c* oxidase. Importantly, the majority of both proteins associated with other complexes or were available in the free form (Figure 1C). Upon loss of Rcf1, *bc*_1_ complex shifted partly out of the higher supercomplex into the lower supercomplex and monomeric form (indicated by Cor2). The same shift was observed for the cytochrome *c* oxidase (Cox2, Cox12, and Cox13) indicating that in the absence of Rcf1 supercomplexes can still be formed, but not as efficient as in the presence of Rcf1. Interestingly, in the absence of Rcf1, major parts of Rcf2 shift from the supercomplexes and cytochrome *c* oxidase into smaller complexes or the free form. Lack of Rcf2 did not affect supercomplex or cytochrome *c* oxidase assembly, or the interaction of Rcf1 with these complexes. Absence of both proteins, Rcf1 and Rcf2, resembled the loss of Rcf1. These results showed that Rcf1 and Rcf2 are not essential for supercomplex formation, but affect the ability of the individual complexes to assemble into supercomplexes.

### Rcf1 and Rcf2 Affect Cytochrome *c* Oxidase Activity in an Opposing Manner

**FIGURE 2:**
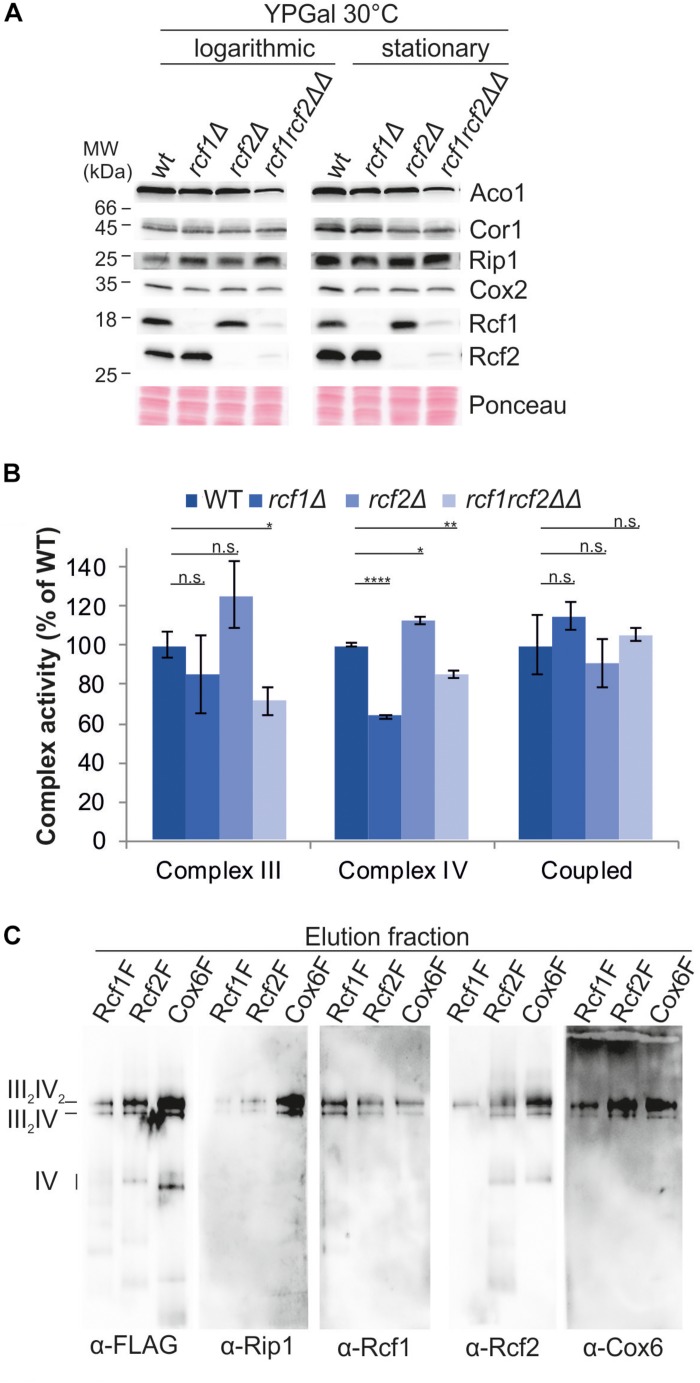
Rcf1 and Rcf2 affect cytochrome *c* oxidase activity without affecting its subunit amount. **(A)** Steady state protein levels of cells grown to logarithmic or stationary phase showed that the loss of Rcf1 or Rcf2 did not change the protein levels of *bc*_1_ complex (Cor1, Rip1) or cytochrome *c* oxidase subunits (Cox2). As control aconitase (Aco1) levels and total protein amount (ponceau) were used. **(B)** Cytochrome *c* reduction measurements showed that only in absence of both Rcf1 and Rcf2 *bc*_1_ complex (Complex III) activity was decreased. Oxygen consumption measurements demonstrated that cytochrome *c* oxidase (Complex IV) activity was decreased upon loss of either Rcf1 or both Rcf1 and Rcf2 while loss of Rcf2 alone led to an increase in activity. The coupled activity of the supercomplexes (Coupled) was not affected by the absence of either Rcf1 or Rcf2 [the standard errors were calculated from a sample size of *n* = 3 (complex III and complex IV) or *n* = 4 (coupled)]. n.s., *P* > 0.05; ^∗^*P* ≤ 0.05; ^∗∗^*P* ≤ 0.01; ^∗∗∗^*P* ≤ 0.001; ^∗∗∗∗^*P* ≤ 0.0001. **(C)** Supercomplexes were purified from mitochondria (cells grown YPG, 30°C, to logarithmic phase) via a FLAG-tag on Rcf1 (Rcf1F), Rcf2 (Rcf2F) or the cytochrome *c* oxidase subunit Cox6 (Cox6F). All three proteins co-purified supercomplexes but to different amounts.

To investigate impact of the Rcf proteins on the individual complexes, we analyzed *bc*_1_ complex and cytochrome *c* oxidase separately. Steady-state protein levels in cells grown to exponential or stationary phase, respectively, showed no differences in the protein levels of Rip1 and Cor1 (*bc*_1_ complex) or Cox2 (cytochrome *c* oxidase; Figure 2A). Furthermore, the protein levels of Rcf1 and Rcf2 were not affected by the absence of the respective other protein. Since no difference in steady-state levels of the individual complexes was observed, the activity of the complexes or the coupled activity of the supercomplexes might be affected.

To explore the influence of Rcf1 and Rcf2 on the activity of the respiratory chain complexes, we measured cytochrome *c* reduction to determine the specific activity of the *bc*_1_ complex and oxygen consumption to determine the specific activity of cytochrome *c* oxidase (Figure 2B). The activity of the *bc*_1_ complex was unchanged in the absence of either Rcf1 or Rcf2 alone, but absence of both proteins led to ∼30% loss of activity. Cytochrome *c* oxidase activity was affected by the absence of the single proteins. While loss of Rcf1 led to a ∼35% decrease of the O_2_-reduction activity of cytochrome *c* oxidase compared to the wild type enzyme, loss of Rcf2 led to an increase in activity of ∼15%. Surprisingly, loss of both Rcf1 and Rcf2 led to a 15% decrease in activity compared to 30% loss in activity upon absence of Rcf1 alone. To determine the coupled activity of the supercomplexes, oxygen consumption of digitonin solubilized mitochondria was measured. Electrons were supplied to the *bc*_1_ complex by reduced decylubiquinone (DQH_2_). Interestingly, the coupled activity of *bc*_1_ complex and cytochrome *c* oxidase was not affected by Rcf1 or Rcf2.

Our analyses, together with previously published data, showed that Rcf1 and Rcf2 are part of the supercomplexes, and overexpressed Rcf1 was co-purified with supercomplexes (Strogolova et al., 2012; Vukotic et al., 2012). To investigate if Rcf1 and Rcf2 are also co-purified with supercomplexes under endogenous expression levels, supercomplexes were purified via FLAG-tag on Rcf1, Rcf2 or the cytochrome *c* oxidase subunit Cox6 under mild conditions (2% digitonin) to ensure intact supercomplexes (Figure 2C). As expected, supercomplexes can be co-purified with Rcf1 and Rcf2. Strikingly, independent on the protein tagged for purification, both proteins, Rcf1 and Rcf2, were present in the supercomplexes. These data showed that supercomplexes can interact simultaneously with Rcf1 and Rcf2. Nevertheless, purification via Rcf1-FLAG yielded in lower co-purification of Rcf2 and vice versa indicating that Rcf1 and Rcf2 not always bind simultaneously to supercomplexes.

### Rcf1 and Rcf2 Are No Stoichiometric Subunits of Cytochrome *c* Oxidase

**FIGURE 3:**
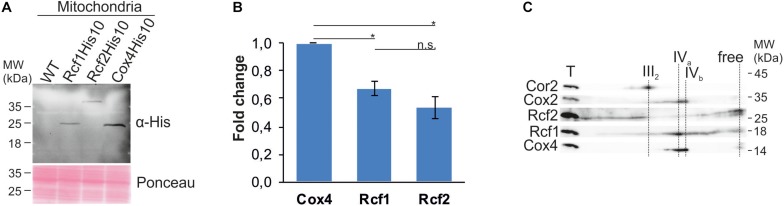
Rcf1 and Rcf2 are no stoichiometric subunits of cytochrome *c* oxidase. **(A)** and **(B)** Steady-state protein levels of His-tagged Rcf1, Rcf2 and the cytochrome *c* oxidase subunit Cox4. Decoration against the epitope of the His-tag showed 30% lower protein levels of Rcf1 and Rcf2 compared to Cox4. The proteins were extracted from mitochondria purified from cells grown in YPG, 30°C. The Western blot signal was quantified using the software ImageJ (the standard errors were calculated from a sample size of *n* = 3). **(C)** BN-PAGE analysis followed by 2D SDS-PAGE, Western blot and immuno decoration of wild-type mitochondria solubilized in 2% DDM showed that Cox4 solely comigrated with cytochrome *c* oxidase, while Rcf1 and Rcf2 comigrated additionally with other complexes. T, total; III_2_, dimer of *bc*_1_ complex; IV_*a*_ and IV_*b*_, forms of cytochrome *c* oxidase. n.s., *P* > 0.05; ^∗^*P* ≤ 0.05; ^∗∗^*P* ≤ 0.01; ^∗∗∗^*P* ≤ 0.001; ^∗∗∗∗^*P* ≤ 0.0001.

These results, together with earlier proposals, raised the question if Rcf1 and Rcf2 are stoichiometric subunits of cytochrome *c* oxidase. However, co-fractionation or co-purification are likely influenced by the solubilization conditions, such that a detergent can dissolve labile bindings of membrane proteins. Hence, we aimed to compare the steady state level of Rcf1 and Rcf2 to those of cytochrome *c* oxidase. Analysis of strains endogenously expressing Rcf1His10, Rcf2His10, and Cox4His10, respectively, showed that Rcf1 and Rcf2 are less abundant than Cox4, a subunit of cytochrome *c* oxidase (Figures 3A,B). Furthermore, analysis by BN-PAGE and subsequent denaturing 2D polyacrylamide gels followed by Western blotting and immuno decoration showed that the majority of Cox4 was assembled in cytochrome *c* oxidase, while Rcf1 as well as Rcf2 were migrating with cytochrome *c* oxidase as well as with other complexes (Figure 3C). Therefore, we concluded that neither Rcf1 nor Rcf2 are stoichiometric subunits of cytochrome *c* oxidase.

### Rcf1 – Supercomplex Association Is Dependent on Carbon Source

**FIGURE 4:**
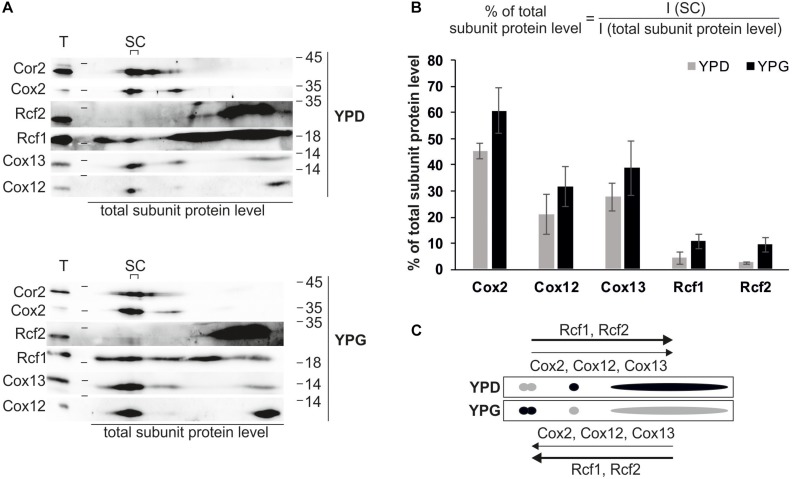
Rcf1 and Rcf2 associate with supercomplexes depending on working load of respiratory chain. **(A)** Mitochondria isolated from cells grown in fermentable (YPD) or non-fermentable medium (YPG) were solubilized in 2% digitonin, separated using BN-PAGE followed by 2D SDS-PAGE, Western blot and immuno decoration. **(B)** Based on the 2D analysis, the amount of the respective subunit of cytochrome *c* oxidase or the amount of the proteins Rcf1 and Rcf2 residing in the supercomplexes was calculated in respect to the total subunit amount. While the cytochrome *c* oxidase subunits slightly shift into the supercomplexes (fold change of ∼1.4), Rcf1 and Rcf2 shift strongly into supercomplexes (∼2.5 and ∼3.7, respectively). The Western blot signals were analyzed using the software OriginPro (the standard errors were calculated from a sample size of *n* = 3). **(C)** Model of protein distribution due to different carbon sources. Under fermentable conditions (YPD) cytochrome *c* oxidase subunits (Cox2, Cox12, and Cox13) are shifted to the monomeric complex or a free form while under respiratory conditions (YPG) cytochrome c oxidase shifts into supercomplexes. Rcf1 and Rcf2 follow the trend but in a higher amount. T, total; SC, supercomplexes; I, intensity of developed western blot.

The association of Rcf1 and Rcf2 with supercomplexes combined with the opposing effects of Rcf1 and Rcf2 on cytochrome *c* oxidase activity raised the question whether these interactions are dependent on the working load of the respiratory chain. To address this question, we isolated mitochondria from cells grown on fermentative medium (YPD) and non-fermentative medium (YPG), separated complexes via BN-PAGE and analyzed co-migration of Rcf1 and Rcf2 with supercomplexes on 2D polyacrylamide gels followed by Western blotting and immuno decoration (Figure 4). As expected, mitochondria grown on respiratory medium showed a higher protein level of respiratory chain complexes than mitochondria grown on fermentable medium. Supercomplexes as well as monomeric cytochrome *c* oxidase were identified on both carbon sources (indicated by Cox2, Cox12, and Cox13), but a clear shift from cytochrome *c* oxidase to supercomplexes was observed under respiratory conditions. Additionally, Rcf1 and Rcf2 shifted into supercomplexes. Since Rcf1 as well as Rcf2 interact with monomeric cytochrome *c* oxidase, the combined shift was expected. Nevertheless, comparatively more Rcf1 (fold change of ∼2.5) and Rcf2 (∼3.7) shifted into supercomplexes than cytochrome *c* oxidase (∼1.4) under respiration. This result indicates that the Rcf proteins are recruited to cytochrome *c* oxidase under growth conditions, in which the respiratory chain has a higher workload.

### The Rcf1 Hig-Domain Is Sufficient to Perform Rcf1 Function

**FIGURE 5:**
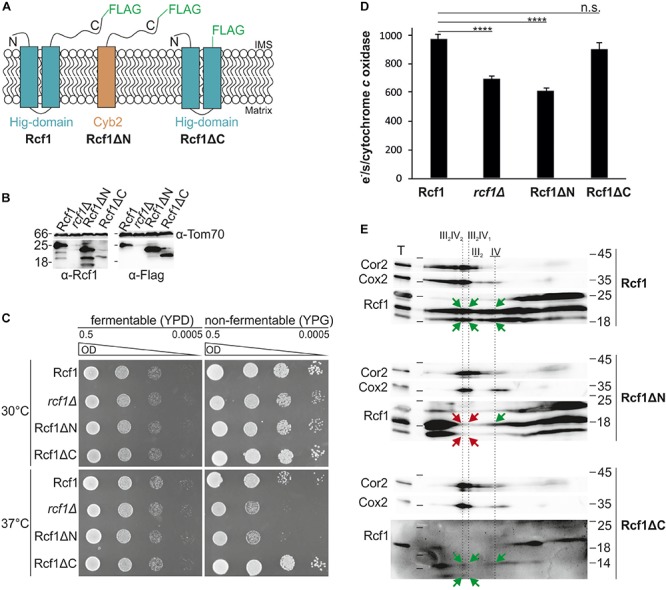
The conserved Hig-domain of Rcf1 fulfills Rcf1 function. **(A)** Schematic view of truncation constructs tagged with a FLAG-tag: full-length Rcf1 (Rcf1), Rcf1ΔN (C-terminus of Rcf1 fused to Cyb2 membrane domain) and Rcf1ΔC (Hig-domain). **(B)** Steady state protein levels of strains expressing full-length Rcf1FLAG (Rcf1; 25 kDa), Rcf1ΔN (23 kDa), Rcf1ΔC (20 kDa) or no Rcf1 (*rcf1*Δ). Mitochondria were solubilized and analyzed using SDS-PAGE followed by Western blot and immunodecoration against Rcf1 and FLAG-peptide. Decoration against Rcf1 shows degradation products which are not visible by decorating against the FLAG-peptide indicating degradation of the FLAG-tag and not the Rcf1-protein itself. **(C)** A 10-fold dilution series of strains grown in YPD to logarithmic phase was spotted on YPD and YPG plates and incubated at 30°C and 37°C. Loss of the Hig-domain (Rcf1ΔN) induces a similar growth defect as loss of the entire protein while loss of the fungi specific C-terminus (Rcf1ΔC) does not affect growth. This growth phenotypes indicate that the conserved Hig-domain (present in Rcf1ΔC) is sufficient to support respiratory growth. **(D)** Oxygen consumption measurements to determine activity of cytochrome *c* oxidase activity in truncated mutants. As expected from the growth assay, loss of the Hig-domain (Rcf1ΔN) led to almost 40% decrease in activity, similar to *rcf1*Δ, while loss of the C-terminus (Rcf1ΔC) showed no effect on the complex activity. **(E)** Truncated mutants were analyzed with BN-PAGE and subsequent 2D SDS-PAGE followed by Western blot and immuno decoration against Cor2, Cox2, and Rcf1. While the C-terminus (Rcf1ΔN) can only comigrate weakly with cytochrome *c* oxidase (green arrow) but not with the supercomplexes (red arrows), the Hig-domain (Rcf1ΔC) retains the function to interact with cytochrome *c* oxidase as well as supercomplexes (green arrows). T, total; III_2_IV_2_, III_2_IV, forms of supercomplexes; III_2_, dimer of *bc*_1_ complex; IV, cytochrome *c* oxidase. n.s., *P* > 0.05; ^∗^*P* ≤ 0.05; ^∗∗^*P* ≤ 0.01; ^∗∗∗^*P* ≤ 0.001; ^∗∗∗∗^*P* ≤ 0.0001.

Rcf1 consists of the conserved Hig-domain and a fungi-specific C-terminus. To identify the function of the individual domains, we constructed strains expressing either full length Rcf1 (Rcf1), only the conserved Hig-domain without the C-terminus (Rcf1ΔC) or only the proposed soluble C-terminus including a mitochondrial targeting signal and a membrane anchor (Rcf1ΔN; Figure 5A). All Rcf1 variants were tagged with a FLAG-tag. Steady state analysis of the Rcf1 variants showed that truncation of the C-terminus led to reduced levels of Rcf1ΔC, while Rcf1ΔN was expressed similar to wild type levels (Figure 5B). Use of the antibody against Rcf1 showed degradation products with the same pattern in Rcf1 full-length and truncation variants. These degradation products were not visible using the antibody against the FLAG-peptide. Therefore, the degradation products most likely originated from degradation of the FLAG-tag and not from the Rcf1 protein itself. Next, we monitored the respiratory ability of the strains performing a growth test at normal conditions (30°C) and at stress conditions (37°C). As expected from former experiments (Figure 1A), the growth phenotype in the absence of Rcf1 at normal conditions was minor, while at 37°C a strong growth defect was observed in the absence of Rcf1 as well as in the presence of only the C-terminus of Rcf1 (Rcf1ΔN). Interestingly, comparably low amounts of Rcf1ΔC were sufficient to sustain normal respiratory growth even during stress conditions (Figure 5C) indicating that the conserved Hig-domain is sufficient to perform the function of the full-length protein. Next, we analyzed the impact of the different domains on the activity of cytochrome *c* oxidase (Figure 5D). In line with the growth phenotype, Rcf1ΔN caused a similar loss of activity as observed in the absence of Rcf1, while cells expressing Rcf1ΔC showed almost the same activity as if full-length Rcf1 is expressed. Comigration assays showed that Rcf1ΔN lost the ability to interact with supercomplexes, while small amounts still comigrate with cytochrome *c* oxidase (Figure 5E; green arrows indicate comigration, red arrows show loss of comigration). On the other hand, even at overall lower protein levels, Rcf1ΔC still comigrated with supercomplexes as well as cytochrome *c* oxidase. These results indicate that small amounts of the Hig-domain (Rcf1ΔC) are sufficient to perform the molecular function of Rcf1, allowing to sustain respiratory growth even under stress conditions.

### Rcf1 Interacts With Cox3

**FIGURE 6:**
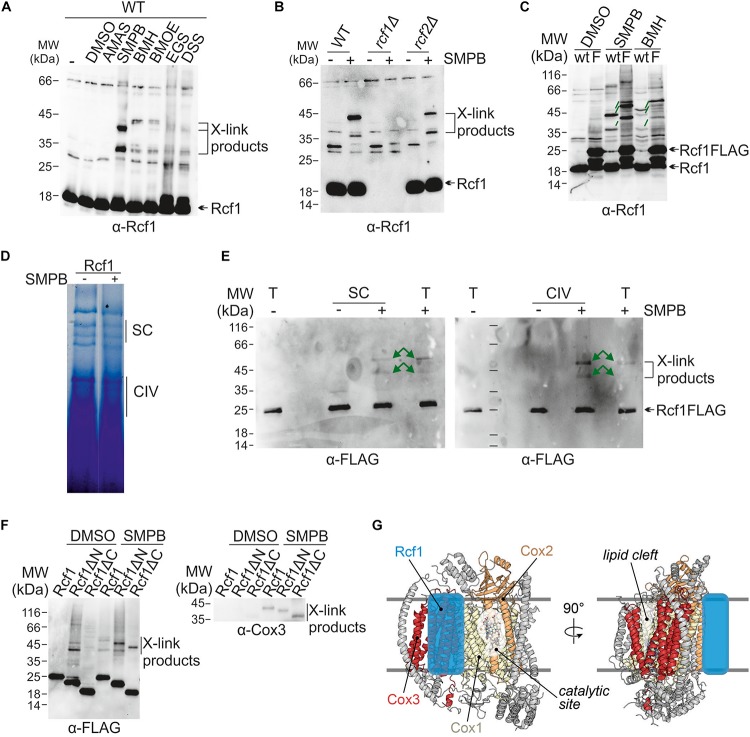
Interaction partners and interaction sites of Rcf1. **(A)** Proteins in purified mitochondria were chemically crosslink followed by protein extraction, SDS-PAGE, Western blotting and immuno decoration against Rcf1. Use of different crosslink reagents (differences in reactive group and length) showed that Rcf1 has multiple interaction partners. The controls (-, DMSO) show cross reactions of the Rcf1 antiserum, which was considered during analysis in this and the following blots. **(B)** Chemical crosslink studies were performed as described in **(A)** with the crosslink reagent SMPB in the presence and absence of either Rcf1 or Rcf2 showed that the crosslink products were independent of Rcf2. **(C)** Chemical crosslink studies of Rcf1 or Rcf1FLAG with SMPB and BMH showed that all crosslink products of Rcf1 shift only 6 kDa (green lines), indicating that none of the crosslink products are self-crosslinks of a Rcf1 dimer. **(D)** Crosslinked (SMPB “+”) and non-crosslinked (SMPB “–“) mitochondria were solubilized in 2% digitonin and separated using BN-PAGE. The pattern of the supercomplexes was not affected by the crosslink. **(E)** As indicated in **(D)** gel samples containing either supercomplexes (SC) or cytochrome *c* oxidase (CIV) as well as total mitochondria fraction (T) were loaded on a polyacrylamide gel followed by transfer to a nitrocellulose membrane and immuno decoration. The crosslink pattern shows the same crosslink products in supercomplexes as in cytochrome *c* oxidase (green arrows). **(F)** Chemical crosslink studies with SMPB (DMSO as control) using the truncation mutants show interactions of both N- and C-termini with Cox3. The antibody against the FLAG-peptide showed unspecific binding in the control (DMSO), which was considered during analysis. **(G)** Model of Rcf1 binding to Cox3 of cytochrome *c* oxidase (adapted from PDB ID: 6HU9). We propose that Rcf1 either directly modulates the active site in Cox1, which is adjacent to Cox3, or Rcf1 modulates the lipid cleft and, thus, the active site through interaction with TM helices of Cox3.

Previous studies showed an interaction between Rcf1 and Cox3 during assembly (Su et al., 2014; Garlich et al., 2017). Therefore, we asked whether this interaction persists in fully assembled cytochrome *c* oxidase. We employed chemical crosslinking to follow these interactions. Purified mitochondria were incubated with a range of chemical crosslinking reagents, varying in length and reactivity. Proteins were extracted and separated via SDS-PAGE followed by Western blotting and immuno decoration to analyze the crosslink products. This approach yielded a range of crosslink products with Rcf1 (Figure 6A). These products neither contained Rcf2 nor were they dependent on the presence of Rcf2 (Figure 6B). To exclude that the crosslink products detected were crosslinks within a potential Rcf1 dimer (Zhou et al., 2018), we compared wt and Rcf1FLAG-tagged strains using the chemical crosslink approach (Figure 6C). In this way, a shift of the crosslink product by 12 kDa (2 × FLAG-tag) was expected for a Rcf1 dimer in the tagged strain. Since only a shift of 6 kDa was observed, no crosslink product was a dimer of Rcf1FLAG. The strongest crosslink products were detected using the crosslink reagent SMPB [succinimidyl 4-(p-maleimidophenylbutyrate)]. Next, we asked whether the detected crosslink products were specific for association of Rcf1 to cytochrome *c* oxidase or supercomplexes. Therefore, we used mitochondria expressing Rcf1FLAG (Rcf1), chemically crosslinked the proteins and separated the complexes using BN-PAGE (Figure 6D). The regions containing cytochrome *c* oxidase or supercomplexes were excised and separated on SDS-PAGE, followed by Western blotting and immuno decoration. The observed crosslink pattern was identical for cytochrome *c* oxidase and supercomplexes indicating that Rcf1 binds to the same sites when cytochrome *c* oxidase is free or associated in supercomplexes (Figure 6E).

As next step contact sites of Rcf1 with Cox3 were established using the strains expressing either full-length Rcf1 (Rcf1), only the Hig-domain (Rcf1ΔC) or only the C-terminus (Rcf1ΔN). All proteins were tagged with a FLAG-tag. Using this approach, we found that the cytochrome *c* oxidase subunit Cox3 interacts with Rcf1 (Figures 6F,G), in line with previous results (Strogolova et al., 2012; Su et al., 2014). Surprisingly, both the Hig-domain and the C-terminus interacted individually with Cox3 indicating a strong interaction with the full-length protein.

## Discussion

**FIGURE 7:**
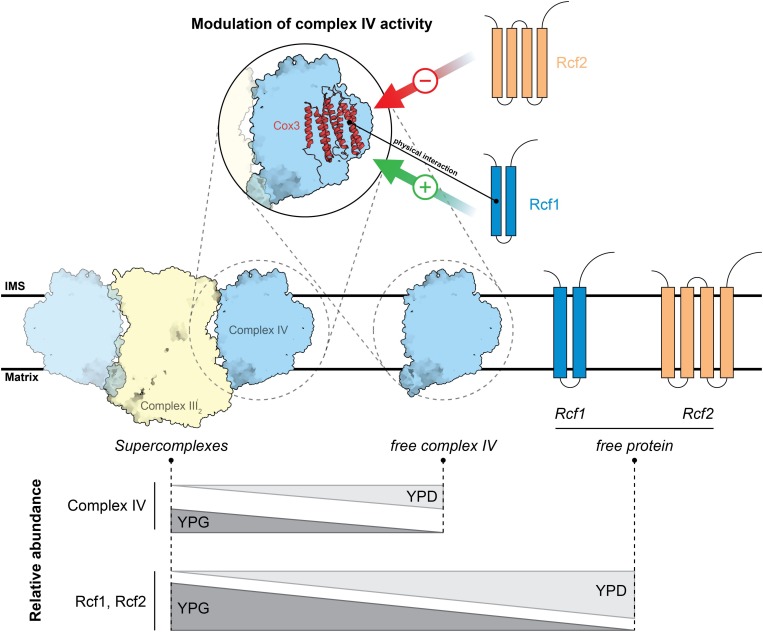
Model of Rcf1 and Rcf2 modulating cytochrome *c* oxidase upon metabolic changes. Rcf1 positively modulates cytochrome *c* oxidase activity while Rcf2 has a negative effect. Upon a high working load of the respiratory chain under non-fermentable conditions (YPG) Rcf1 and Rcf2 shift overproportional into supercomplexes compared to a slight shift of cytochrome *c* oxidase subunits. Therefore, we speculate that the respiratory chain must be tightly regulated or has higher need of repair under a high working load. Structures are adapted from PDB ID: 6HU9.

The supramolecular assembly of respiratory chain complexes to form supercomplexes and respirasomes has fueled intensive research into the function and biogenesis of these structures. Rcf1 and Rcf2 have been identified as components of the supercomplexes in yeast, comprised of the *bc*_1_ complex and cytochrome *c* oxidase. Several functions for Rcf1 and Rcf2 have been proposed, ranging from assembly factors for supercomplexes to being subunits of cytochrome *c* oxidase (Chen et al., 2012; Strogolova et al., 2012; Vukotic et al., 2012; Rydström Lundin et al., 2016; Garlich et al., 2017; Schäfer et al., 2018a). Here, we studied the physiological significance of Rcf1 and Rcf2 and their molecular interactions as they occur in mitochondria (Figure 7). It was initially proposed that Rcf1 and Rcf2 are subunits of cytochrome *c* oxidase (Vukotic et al., 2012). However, we showed that the amount of supercomplexes purified via Rcf1FLAG or Rcf2FLAG was lower than when purified via a cytochrome *c* oxidase subunit (Cox6FLAG). These data combined with deletion studies (Figures 1B,C) indicate that subpopulations of supercomplexes exist, a population interacting with Rcf1 and Rcf2 simultaneously and other subpopulations that interact separately with Rcf1 or Rcf2 or lack any Rcf protein. We furthermore showed the lower steady-state protein levels of Rcf1 and Rcf2 compared to cytochrome *c* oxidase subunit Cox4. Moreover, Cox4 almost exclusively assembles into the cytochrome *c* oxidase complex, while Rcf1 and Rcf2 associate, in addition to cytochrome *c* oxidase, also with other protein complexes and can even exist in free forms. These Rcf1-containing complexes are established through molecular interaction of Rcf1 with Cox3, Aac2 (Strogolova et al., 2012) and likely other proteins. Results reported here demonstrate that both the conserved Hig-domain encompassing two transmembrane helices as well as the yeast-specific C-terminal domain participate in these interactions.

As described previously (Chen et al., 2012; Strogolova et al., 2012; Vukotic et al., 2012), Rcf1 and Rcf2 comigrate with supercomplexes and cytochrome *c* oxidase. Importantly, supercomplex formation was only slightly affected in the absence of Rcf1 and the combined loss of Rcf1 and Rcf2, but not affected in the absence of only Rcf2. Furthermore, we showed that the cytochrome *c* oxidase subunits Cox12 and Cox13 still assemble into cytochrome *c* oxidase as well as supercomplexes, which was not observed previously (Strogolova et al., 2012; Vukotic et al., 2012). However, the mild assembly defects of supercomplexes in the absence of Rcf1 cannot explain the growth defect in absence of Rcf1 or both Rcf1 and Rcf2.

To further investigate the impact of loss of the Rcf proteins on respiration, we analyzed expression levels and activities of the individual complexes. Absence of either Rcf1 or Rcf2 did not affect levels of *bc*_1_ complex subunits, cytochrome *c* oxidase subunits or the respective other protein. However, loss of Rcf1 led to a severe decrease in cytochrome *c* oxidase activity, while loss of Rcf2 led to an increase. These results indicate that Rcf1 and Rcf2 may function as modulators of cytochrome *c* oxidase, where Rcf1 stimulates the enzyme, while Rcf2 inhibits its activity. In this way, cytochrome *c* oxidase may react to different energy demands within the cell. In line with our findings, HIGD1A, a mammalian homolog of Rcf1, was shown to be a modulator of the cytochrome *c* oxidase in neonatal rat cardiomyocytes (Hayashi et al., 2015). Earlier studies showed that in the absence of Rcf1, different populations of cytochrome *c* oxidase exist (Rydström Lundin et al., 2016; Garlich et al., 2017; Schäfer et al., 2018a, b), namely a population with fully functional cytochrome *c* oxidase and a population with a less active, structurally impaired enzyme. Furthermore, Rcf1 and Rcf2 are not stoichiometric subunits of cytochrome *c* oxidase as shown in this study. Since we determine the activity of the entire population these measurements underestimate the effect of the absence of Rcf1 or Rcf2 on the afflicted subpopulation of cytochrome *c* oxidases. We propose that Rcf1 modulates the activity by either binding to monomeric cytochrome *c* oxidase or when the protein is found in respiratory supercomplexes. To ensure optimal respiration, the cell might be able to steer only the fully functional oxidase population into supercomplexes. However, oxidase can still accumulate in the absence of Rcf1, but a significant fraction is structurally changed (Schäfer et al., 2018b).

How could Rcf1 modulate oxidase function? We hypothesize that Rcf1, by binding to Cox3, can modulate the active site found in Cox1 in two principal ways. First, Rcf1 could directly interact with the TM helices of Cox1 around the catalytic site, influencing their structure. This is in line with a previously described structural change in the environment around the catalytic site (Rydström Lundin et al., 2016; Schäfer et al., 2018a, b). Alternatively, Rcf1 could bind to the TM helices of Cox3 to induce structural changes in the lipid cleft. According to the yeast supercomplex structure (Hartley et al., 2019), this cleft contains phosphatidylethanolamine. Furthermore, Varanasi et al. (2006) showed an interaction between the active site (Cu_*B*_) and the lipids residing in the clefts in *Rhodobacter sphaeroides*. Consequently, Rcf1 could change the environment of PE in a way that the activity of cytochrome *c* oxidase changes. Interestingly, in yeast, Cox3 is positioned on the opposite site of the supercomplex interface consisting of Cox5 and Cor1 (Hartley et al., 2019; Rathore et al., 2019), questioning the earlier proposed function of Rcf1 to physically mediate supercomplex formation (Chen et al., 2012; Strogolova et al., 2012; Vukotic et al., 2012; Rydström Lundin et al., 2016). Moreover, Rcf1 and Rcf2 preferentially associate with supercomplexes under respiratory growth conditions. This might indicate that in a state when the cell depends on respiration and the working load for the respiratory chain is high, Rcf1 and Rcf2 interact with subpopulations of cytochrome *c* oxidase to maintain the protein in a functional state.

## Materials and Methods

### Strain Construction

All *S. cerevisiae* strains (Supplementary Table 1) were isogenic to BY4741 (MATa; ura3Δ0; leu2Δ0; his3Δ1; met15Δ) except of strains containing His-tagged proteins which were isogenic to W303a (MATa; leu2-3,112 trp1-1 can1-100 ura3-1 ade2-1 his3-11,15). The *rcf1*Δ and *rcf2*Δ were acquired from euroscarf. To construct *rcf1rcf2*ΔΔ the RCF1 gene in *rcf2*Δ was replaced by a *URA3* cassette using homologous recombination. All proteins except of Rcf1 were FLAG-tagged genomically using homologous recombination. The FLAG-tag including a *URA3* selection cassette was amplified from pYM28-FLAG. All proteins were His-tagged genomically. The His10-tag including a *TRP1* selection cassette was amplified from pYM29-His10 (Supplementary Table 2). The integrative plasmid pRS403 Rcf1FLAG and its truncated versions (see plasmid construction) were integrated into the *rcf1*Δ strain to express Rcf1FLAG and Rcf1ΔC under its endogenous promoter while _*C*_Rcf1ΔN was expressed under Cyb2 promoter.

### Plasmids

To construct pRS403 Rcf1FLAG, first, the FLAG-tag including a linker region and a terminator was amplified from pYM28-FLAG and cloned into pRS403 using the restriction sites *Eco*RI and *Xho*I. Subsequently, Rcf1 including the endogenous promoter but missing the stop codon was amplified from gDNA (BY4741 wt) and inserted into the plasmid using the restriction sites *Not*I and *Eco*RI. To construct Rcf1ΔC the promoter region and part of Rcf1 (residues 1 to 95) was amplified and from gDNA (BY4741 wt) and cloned into pRS403-FLAG using *Not*I and *Eco*RI. To construct Rcf1ΔN only part of Rcf1 was amplified (residues 86 to 159) and cloned into pRS403-FLAG with the restriction enzymes *Sma*I and *Eco*RI. As membrane anchor the N-terminal membrane domain of Cyb2 (residues 1 to 76) including its promoter was amplified and inserted into pRS403-Rcf1ΔN_FLAG with the restriction enzymes *Not*I and *Sma*I. Primers are listed in Supplementary Table 2.

To integrate the plasmid into the yeast genome it was cut within the HIS gene with *Nhe*I.

### Growth Analysis

To analyze the respiratory capacity of strains lacking Rcf1, Rcf2 or both proteins, strains were grown in YPD (1% yeast extract, 2% peptone, 2% glucose) at 30°C to logarithmic phase and cells were harvested at OD_600_ 0.5. A 10-fold serial dilution was spotted on synthetic medium plates containing glucose or glycerol (0.17% yeast nitrogen base, 0.5% ammonium sulfate, all amino acids, 2% glucose or 2% glycerol, respectively). The plates were incubated at 25°C, 30°C, and 37°C. The same procedure was followed for strains expressing full-length Rcf1 or truncated mutants except cells were spotted on YPD and YPG plates (1% yeast extract, 2% peptone supplemented with 2% glucose or 2% glycerol).

### Mitochondrial Isolation

Strains were grown in YPD/YPGal/YPG (1% yeast extract, 2% peptone, 2% glucose/2% galactose/2% glycerol) to logarithmic phase, harvested (5 min, 4000 rpm), washed with dH_2_O and incubated in MP1 buffer (2 ml/g cell wet weight; 0.1 M Tris–base, 10 mM dithiothreitol) for 10 min at 30°C. Subsequent washing of the cells (1.2 M sorbitol) and incubating in zymyolase buffer (6.7 ml/g cell wet weight; 20 mM KPi pH 7.4, 0.6 M sorbitol, 3 mg/g cell wet weight zymolyase 20T [Seikagaku Biobusiness, Tokyo, Japan]) for 60 min at 30°C yielded in spheroplasts. Spheroplasts were harvested, resuspended in homogenization buffer (13.4 ml/g cell wet weight; 10 mM Tris pH 7.4, 0.6 M sorbitol, 1 mM EDTA, 1 mM PMSF) and homogenized (teflon plunger in a tight fitting homogenizer (Sartorius Stedim Biotech S.A., Aubagne, France). Using differential centrifugation first cell debris was separated from the supernatant containing mitochondria (5 min, 4000 rpm) to then harvest mitochondria (15 min, 10 000 *g*). Mitochondria were resuspended in isotonic buffer (0.6 M Sorbitol, 20 mM HEPES pH 7.4; 10 mg/ml), shock frozen in liquid nitrogen and stored at −80°C.

### BlueNative-PAGE Analysis Including Subsequent Western Blot or 2D SDS-PAGE

Mitochondria were resuspended in solubilization buffer (2% digitonin/DDM = 2 mg digitonin/DDM per mg protein) and incubated on ice for 10 min. The samples were centrifuged (7 min, 25 000 *g*), the supernatant was saved and mixed with 0.5% sample additive (NativePAGE^TM^ 5% G-250 Sample Additive; Thermo Fisher Scientific). The samples were loaded (100 μg per sample) on a 3–12% NativePage Bis-Tris gel (Thermo Fisher Scientific) using NativePAGE^TM^ 1X Running Buffer as anode buffer and NativePAGE^TM^ 1X Running Buffer supplemented with 0.02% Coomassie G-250 as cathode buffer (Thermo Fisher Scientific). The gel was run until 1/3 of the gel at 150 mV, then the cathode buffer was exchanged to NativePAGE^TM^ 1X Running Buffer and the gel was run until the end at 250 mV. Subsequently, the gel was either transferred to a PVDF membrane (Bio-Rad; 100° mA, 90° min) or a second dimension denaturing polyacrylamide gel was run. For the second dimension each sample lane was cut out of the gel entirely or partly, incubated 20 min in running buffer containing 100 mM DTT and inserted in a 90° angle in a denaturing polyacrylamide gel (16% acrylamide, 0.2% bisacrylamide; 30 mA, 50 min). The proteins were subsequently transferred to a nitrocellulose membrane (Amersham^TM^ Protran^®^ Premium Western blotting membranes). Antibodies were prepared in 5% milk dissolved in TBS.

### Protein Steady States

Cells were grown in YPGal (1% yeast extract, 2% peptone, 2% galactose) and harvested at exponential or stationary phase. Proteins were extracted using alkaline lysis (0.1 M NaOH incubated 5 min, RT; centrifugation: 14 000 rpm, 2 min). The pellet was resuspended in reducing sample buffer (50 mM Tris–HCl pH 6.8, 2% SDS, 10% glycerol, 100 mM DTT). Samples were loaded on 16% polyacrylamide, 0.2% bisacrylamide gels (30 mA, 50 min) and transferred to nitrocellulose membranes (Amersham^TM^ Protran^®^ Premium Western blotting membranes).

### Enzyme Assays

Mitochondria were resuspended in solubilization buffer (50 mM Tris, 150 mM KCl, 1 mM EDTA, 1x complete, 1 mM PMSF, 2% digitonin) and after a clarifying spin the supernatant was used for the respective experiment.

To measure *bc*_1_ complex activity 5 μg of mitochondrial supernatant was added to 50 mM Tris, 50 μg cytochrome *c*, 1 mM KCN (to block cytochrome *c* oxidase) and 0.24 mM reduced decylubiquinone (DQH_2_). The change in absorbance of cytochrome *c* at 550 nm was monitored. The reaction was stopped by adding 0.36 mM antimycin. The activity was normalized to *bc*_1_ complex content determined by spectral analysis (ε_562__–__575_ = 28.5 mM^–1^ cm^–1^).

Oxygen consumption by cytochrome *c* oxidase was measured using a Clark-type electrode. A baseline was recorded for buffer (50 mM Tris, pH 7.4, 100 mM KCl, 100 μM EDTA) with 5 mM ascorbate, 0.5 mM TMPD. To start the reaction, 250 to 500 μg of lysed mitochondrial supernatant and cytochrome *c* (25 μM) was added. Oxygen consumption rates were normalized to the amount of cytochrome *c* oxidase (ε_603__–__621_ = 26 mM^–1^ cm^–1^).

To measure the coupled activity of *bc*_1_ complex and cytochrome *c* oxidase a baseline was recorded for buffer (50 mM Tris, pH 7.4, 100 mM KCl, 100 μM EDTA) with DQH_2_ (2 μM). Addition of 250 to 500 μg of mitochondrial supernatant and cytochrome *c* (25 μM) started the reaction. Oxygen consumption was measured using a Clark-type electrode. The activity was normalized to the amount of cytochrome *c* oxidase (ε_603__–__621_ = 26 mM^–1^ cm^–1^).

### Chemical Crosslink

Mitochondria were incubated with isotonic buffer (0.6 M Sorbitol, 20 mM HEPES pH 7.4) containing either DMSO as control or the respective crosslink reagent (c_*f*_ = 200 μM) dissolved in DMSO for 30 min at 30°C. The reaction was quenched with either Tris pH8 (c_*f*_ = 100 mM) or β-mercaptoethanol (c_*f*_ = 100 mM) or both depending on the reactive group of the crosslink reagent. The crosslinking products were analyzed directly or after purification on denaturing polyacrylamide gels. The following crosslink reagents were used in this study: SMPB (succinimidyl 4-(p-maleimidophenyl)butyrate), AMAS (N-α-maleimidoacet-oxysuccinimide ester), BMH (bismaleimidohexane), BMOE (bismaleimidoethane), EGS [ethylene glycol bis (succinimidyl succinate)] and DSS (disuccinimidyl suberate). All reagents were purchased from ThermoFisher scientific.

## Data Availability Statement

The datasets generated for this study are available on request to the corresponding author.

## Author Contributions

HD and MO contributed to the conceptualization and the review, editing, and writing of the manuscript. HD, JS, PB, and MO contributed to the methodology. HD and JS contributed to the software, validation, formal analysis, and visualization. HD, JS, JMS, and WM contributed to the investigation. MO and PB contributed to the resources, supervision, and funding acquisition. HD contributed to the data curation and writing of the original draft. HD, MO, and PB contributed to the project administration.

## Conflict of Interest

The authors declare that the research was conducted in the absence of any commercial or financial relationships that could be construed as a potential conflict of interest.
