# Supplementary material for: Rcf1 Modulates Cytochrome c Oxidase Activity Especially Under Energy-Demanding Conditions
Source: Front Physiol. 2020 Jan 14;10:1555. doi: 10.3389/fphys.2019.01555 (PMC6971206; doi:10.3389/fphys.2019.01555)
Supplement: Supplementary file 1 [file Table_1.pdf]

Supplementary Table 1: Strains used in this work.

**Strain name**	**Alias in this work**	**Source**
BY4741	wt	Euroscarf (Y00000)
BY4741 *rcf1*Δ	*rcf1*Δ	Euroscarf (Y00543)
BY4741 *rcf2*Δ	*rcf2*Δ	Euroscarf (Y05393)
BY4741 *rcf1rcf2*ΔΔ	*rcf1rcf2*ΔΔ	This study.
BY4741 *rcf1*Δ pRS403 Rcf1FLAG	Rcf1F/Rcf1	This study.
BY4741 Rcf2FLAG	Rcf2F	This study.
BY4741 Cox6FLAG	Cox6F	This study.
W303a	WT	This study.
W303a Rcf1His10	Rcf1His10	This study.
W303a Rcf2His10	Rcf2His10	This study.
W303a Rcf1His10	Rcf1His10	This study.
BY4741 *rcf1*Δ pRS403 Rcf1ΔN_FLAG	Rcf1ΔN	This study.
BY4741 *rcf1*Δ pRS403 Rcf1ΔC_FLAG	Rcf1ΔC	This study.

Supplementary Table 2: Primers used in this work.

**Strain/Plasmid**		**Sequence 5' – 3'**
*rcf1*Δ in BY4741 *rcf1rcf2*ΔΔ	For	CTGGTACTAGAAGCGATAACATCACAGTTATACGTT CAAGCCAGCTGAAGCTTCGTACGCTGC
	Rev	CTCTCATCATAATTTATTTACAGCAAATCGAAGAGCC AAAATAGGCCACTAGTGGATCTG
BY4741 Rcf2FLAG	For	GGCTACGTTTCTAACGAAGAAAGAATAAACTCCAAG ATCTTCAAGTCGCGTACGCTGCAGGTCGACGG
	Rev	CCGGTTATTGCGCGAGAAGCCGTTAAATGTTAACTCC GCGGGACCGACAAGCATCGATGAATTCGAGCTCG
BY4741 Cox6FLAG	For	GACAAGAATTGGGCGTTCCCTTAAAGGAAGAGCTAT TTCCAAGCTCTTCTCGTACGCTGCAGGTCGAC
	Rev	GTTATTTATTGTTTTATTATTTTTCCCATTCTTCTTTCC TGAAATAGAACATCGATGAATTCGAGCTCG
W303a Rcf1His10	For	CTTAGAAAAGGAACTAAGCGACCTGGAAAATAAGCT TGGAAAGAAGCGTACGCTGCAGGTCGAC
	Rev	GTACCGATCGTTAGCTGGCAATTACCGTTACGTATGT GTCAAGCATCGATGAATTCGAGCTCG
W303a Rcf2His10	For	CCGGCTACGTTTCTAACGAAGAAAGAATAAACTCCA AGATCTTCAAGTCGCGTACGCTGCAGGTCGAC
	Rev	CCGGTTATTGCGCGAGAAGCCGTTAAATGTTAACTCC GCGGGACCGACATCGATGAATTCGAGCTCG
W303a Cox4His10	For	TACAAACTAAACCCTGTTGGTGTTCCAAATGATGACC ACCATCACCGTACGCTGCAGGTCGAC
	Rev	CTTCCTGCAAAGAACTTTCTTGTGGAACTTTGTTAAC ATGACGTGTGATCGATGAATTCGAGCTCG
pRS403 Rcf1FLAG	Rcf1 prom for NotI	GCGGCCGCAGCACCCCTCCTCCCATTTC
	Flagtag+term rev XhoI	CGCTCGAGTCAAGGAGGGTATTCTGGGC
	Spacer Flag S3 for EcoRI	GCGAATTCCGTACGCTGCAGGTCGAC
	promRcf1-stop rev EcoRI	GCGAATTCCTTCTTTCCAAGCTTATTTTC
pRS403 Rcf1ΔN_FLAG	Rcf1 prom for NotI	GCGGCCGCAGCACCCCTCCTCCCATTTC
	Rcf1 Trunc1 EcoRI rev	GCGAATTCCTTCGCCTTCAGTTCCTTACCAG
pRS403 Rcf1ΔC_FLAG	Rcf1 Cterm for SmaI	CGCCCGGGGGGACTTCTGGTAAGGAACTG
	Rcf1 Cterm rev EcoRI	CGCGAATTCCTTCTTTCCAAGCTTATTTTC
	Cyb2 for NotI	CCGCGGCCGCAAGCCTGCCGATATCTCCTT
	Cyb2short rev SmaI	GGCCCGGGGCCATTATGCCAGTTTAGATAC
